# Short-wavelength infrared photodetector on Si employing strain-induced growth of very tall InAs nanowire arrays

**DOI:** 10.1038/srep10764

**Published:** 2015-06-02

**Authors:** Hyun Wook Shin, Sang Jun Lee, Doo Gun Kim, Myung-Ho Bae, Jaeyeong Heo, Kyoung Jin Choi, Won Jun Choi, Jeong-woo Choe, Jae Cheol Shin

**Affiliations:** 1 Korea Photonics Technology and Institute, Gwangju 550-779, Republic of Korea; 2 Department of Applied Physics, Kyung Hee University, Yongin 446-701, Republic of Korea; 3 Korea Research Institute of Standards and Science, Daejeon, 305-340, Republic of Korea; 4 Department of Materials Science and Engineering, and Optoelectronics Convergence Research Center, Chonnam National University, Gwangju 500-757, Republic of Korea; 5 School of Materials Science and Engineering, Ulsan National Institute of Science & Technology, Ulsan 689-805, Republic of Korea; 6 Korea Institute of Science and Technology, Seoul 136-791, Republic of Korea; 7 Department of Physics, Yeungnam University, Gyeongsan, Gyeongbuk 712-749, Republic of Korea

## Abstract

One-dimensional crystal growth enables the epitaxial integration of III-V compound semiconductors onto a silicon (Si) substrate despite significant lattice mismatch. Here, we report a short-wavelength infrared (SWIR, 1.4–3 μm) photodetector that employs InAs nanowires (NWs) grown on Si. The wafer-scale epitaxial InAs NWs form on the Si substrate without a metal catalyst or pattern assistance; thus, the growth is free of metal-atom-induced contaminations, and is also cost-effective. InAs NW arrays with an average height of 50 μm provide excellent anti-reflective and light trapping properties over a wide wavelength range. The photodetector exhibits a peak detectivity of 1.9 × 10^8^  cm·Hz^1/2^/W for the SWIR band at 77 K and operates at temperatures as high as 220 K. The SWIR photodetector on the Si platform demonstrated in this study is promising for future low-cost optical sensors and Si photonics.

III-V semiconductors have been successfully utilized as high performance optical devices because of their direct bandgap and bandgap tunability using various alloy systems^1^. Meanwhile, silicon (Si) is the most universally used platform in the electronics industry; thus, integrating III-V semiconductor onto Si can lead to compact optical integrated circuits. For example, conventional semiconductor infrared (IR) imaging sensors are fabricated via a hybrid bonding of the III-V cells onto complementary metal oxide semiconductor (CMOS)-based readout integrated circuits^2^,^3^. However, this bonding process entails significant production costs and limits the size reducibility of the device. Alternatively, an epitaxial integration of a III-V semiconductor onto Si offers unique properties, such as large-scale integration and low-cost production^4^,^5^. Nanowires (NWs) allow for the heterogeneous epitaxy of a III-V semiconductor onto the Si substrate despite their different crystal lattice parameters and thermal expansion behaviors^6^,^7^. Hence, the growth of III-V NWs on Si has been widely researched, and light-emitting diodes (LEDs) consisting of III-V NWs grown on Si have recently been demonstrated to be Si photonics building blocks^8^. A photodetector for short-wavelength infrared (SWIR, 1.4–3 μm) on Si is particularly attractive for Si photonics because Si itself is an excellent waveguide with very low loss across the SWIR band^9^. Moreover, detecting SWIR light is useful for many applications such as optical communications, remote sensing and spectral imaging^10^,^11^. In this letter, we demonstrate a SWIR photodetector based on InAs NWs grown on Si. Importantly, wafer-scale densely packed InAs NW arrays were formed on the Si substrate without a metal catalyst or pattern assistance. The optical and electrical properties of these InAs NW arrays were experimentally investigated. The optoelectronic properties of the NW-based photodetector on Si were examined in the SWIR region at various temperatures.

## Results

### Growth of InAs NWs on Si

**Figure 1:**
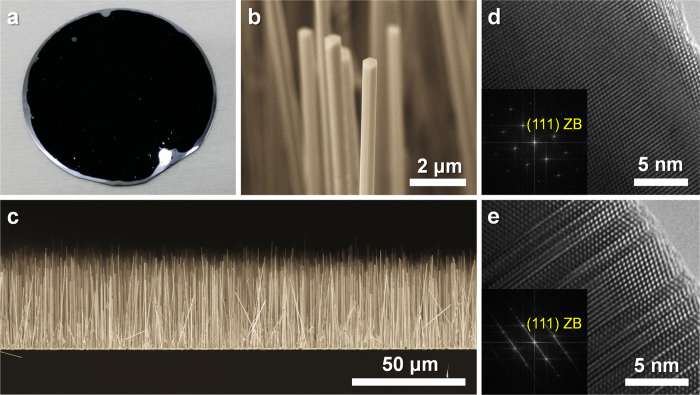
InAs NW array growth on Si (111) substrate. (**a**) Optical image of the InAs NW array grown on a 2-inch Si wafer. (**b**) SEM image tilted at 45° to show that the NWs have hexagonal tips. (**c**) Side-view SEM images showing that most NWs are vertically grown over a large area. (**d, e**) HR-TEM images and corresponding ED patterns (inset).

Figure 1a shows an optical image of InAs NWs grown on a 2-inch Si (111) substrate. Remarkably, the NWs grew uniformly across the entire wafer at a density of approximately 2 × 10^7^ cm^−2^. The black color of the wafer surface suggests that the dense NW array reflects hardly any visible light. The top of the NW is hexagonal as shown in Fig. 1b, which implies a heterogeneous NW growth on the Si (111) surface with a hexagonal closed pack that continues homoepitaxially on the InAs (111)B surface^12^. The side-view scanning electron microscopy (SEM) image in Fig. 1c shows that the NWs have an average height of 50 μm and some of the NWs are slightly tilted. Almost all InAs NWs (>99%) are grown vertically on the Si (111) surface at the beginning of the growth. However, some portion of the NWs is slanted as their height increases (see the Figure S1 of the Supplementary Information). The high-resolution transmission electron microscopy (HR-TEM) image and electron diffraction (ED) patterns shown in Fig. 1d indicate that the InAs NWs exhibit a zinc blende (ZB) crystal structure. Stacking faults along the NW growth direction were randomly observed, as shown in Fig. 1e. Nevertheless, we expect that no threading dislocation exists across the NW because NW geometry can suppress the generation of threading dislocation despite the large lattice-mismatch (i.e., 11.6%)^13^,^14^. For the NW growth, we emphasize the wafer-scale InAs NW array formed via lattice mismatch strain; therefore, we call this a “strain-induced” growth method. The strain-induced growth of InAs NWs on Si was first demonstrated by Wei *et al*^15^. The InAs crystal growth on a Si substrate is started by forming isolated InAs islands on the substrate surface to relieve lattice-mismatch strain from the heterointerface^16^. These islands can preferentially elongate along the <111> direction under proper growth conditions. Meanwhile, hexagonal geometry between the {110} sidewalls and the (111)B facets remains because high energy facets grow out quickly, and only low index facets are left in the growth^17^. We previously reported that the uniformity and density of the InAs NWs increases dramatically when the Si substrate is briefly immersed in a poly-L-lysine solution before the growth because the thin polyelectrolyte layer on the Si surface from the immersion promotes the formation of the isolated islands^18^.

### Optical properties of InAs NWs

**Figure 2:**
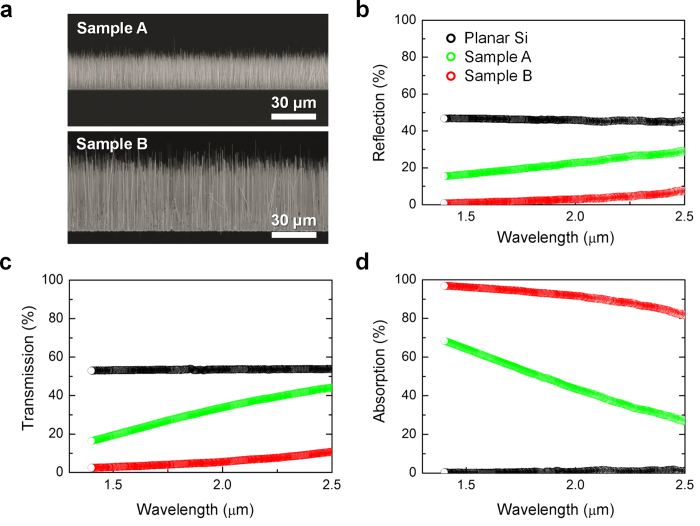
Optical characterization of InAs NW array on Si. (**a**) Side-view SEM images showing the 25- and 50-μm-high NW arrays (Samples A and B, respectively). (**b**) Reflection, (**c**) transmission, and (**d**) absorption spectra of samples A and B with those of a planar Si reference.

The geometry of the NW array provides excellent anti-reflection (AR) properties over a large wavelength range and renders an AR coating unnecessary^19^. Moreover, the NW array enhances the path length of the incident light and reduces the material consumption of the absorbed light relative to conventional planar schemes^20^. The optical properties of the strain-induced InAs NW array were experimentally measured at two different NW heights. Figure 2a shows SEM images for samples A and B, wherein the NW arrays are approximately 25 and 50 μm high, respectively. The average diameters of the NWs for sample A and sample B are 130 nm and 250 nm, respectively, but their densities are nearly the same. The reflection (Fig. 2b) and transmission (Fig. 2c) spectra were measured across the wavelength range from 1.4–2.5 μm for samples A and B, along with a planar Si reference. For the reference sample (i.e., planar Si), nearly 50% of the incident light was reflected by the Si surface. Even in the case of planar InAs surface, more than 20% of incident light is reflected in the SWIR region. This light reflection decreased significantly with increasing InAs NW array height. The reflection rate for sample B was below 10% across the measured wavelengths. This result indicates that the densely packed NW array has extremely efficient AR properties over a large wavelength range. The transmission rate also decreased with increasing NW height because incident light with energy above the InAs band-gap (λ_InAs_ = 3.5 μm) was absorbed by the NWs. Figure 2d shows the experimentally measured absorption (100 - R - T) spectra for samples A and B and the planar Si. As expected, no discernible absorption was found for the planar Si. However, the light absorption increased noticeably with increasing InAs NW array height. The NWs are slanted as the NW height increases, and this partly results in an increase of light absorption because the slanted NWs enlarge the surface area against the incident light. Overall, the absorption rate across the measured wavelength range was 46% and 91% for samples A and B, respectively. Because sample B has thicker and taller NWs, the effective volume of sample B is much larger than that of sample A, resulting in an increase of light absorption. In addition, an increase of NW height further enhances the light absorption because high-aspect-ratio NW array acts as very efficient light scattering centers^20^. Considering that the total InAs volume for sample B per unit area corresponds to a 0.5-μm-thick film, the NW array dramatically enhanced the light absorption rate *via* diffraction and trapping along with its excellent AR property^21^. Here the total volume of InAs NWs was calculated by summing up every volume of the NWs in unit area (See Methods).

### InAs NW Photodetector

**Figure 3:**
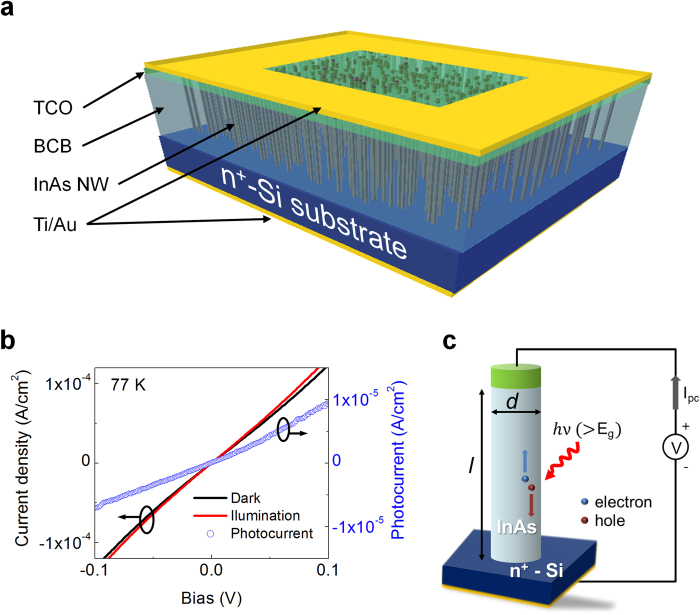
InAs NW array-based photodetector. (**a**) Illustration of the fabricated photodetector. (**b**) Low temperature (77 K) *J-V* characteristics in the dark (*I*_*D*_) and under IR illumination (*I*_*L*_), and the photodetector photocurrent (*I*_*P*_* = I*_*L*_ − *I*_*D*_). (**c**) Illustration of the basic photoconductive-type InAs NW photodetector concept.

Figure 3a illustrates a fully fabricated InAs NW array-based photodetector. A heavily n-doped Si substrate (ρ = 0.002 – 0.005 Ω.cm) and transparent conducting oxide (TCO) are used as the bottom and top electrodes of the InAs NWs, respectively. Then, the Ti/Au contact pads are further deposited. The top planar surface defined by open TCO region is the active area of the photodetector (see the Figure S2 of the Supplementary Information). Figure 3b shows the current density (*J*) - voltage (*V*) characteristics of the photodetector measured at 77 K in darkness (*I*_*D*_) and under IR illumination (*I*_*L*_). The *J - V* curves exhibit Ohmic behaviors across a ± 0.1 V range. The photocurrent (*I*_*P*_* = I*_*L*_ − *I*_*D*_) calculated from the *J - V* curves increased with a bipolar bias, as shown in Fig. 3b. A concept of the photoconductive-type NW photodetector is illustrated in Fig. 3c. When light illuminates the homogeneous NW semiconductor, carriers are generated via an interband transition, which increases the NW conductance^22^. The photoconductor current equation can be expressed as follows:


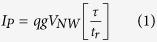


where *q* is the elementary charge, *g* is the photocarrier generation rate, *V*_*NW*_ is the NW volume, *τ* is the minority carrier lifetime, and *t*_*r*_ is the carrier transit time across the NW. The carrier transit time across the NW is given by the following:


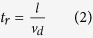


where *l* is the NW length and *ν*_*d*_ is the drift velocity (*ν*_*d*_ = *μE*). Here *μ* and *E* are low-field mobility and electric field, respectively. For InAs, *μ*_*n*_ + *μ*_*p*_ ≈ *μ*_*n*_. The bracketed quantity in equation (1) is called the photoconductive gain (*G*) which is the ratio of the minority carrier lifetime to the carrier transit time^23^.


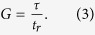


Equation (1) suggests that a long carrier lifetime is essential to a high-performance photoconductive-type photodetector.

## Discussion

**Figure 4:**
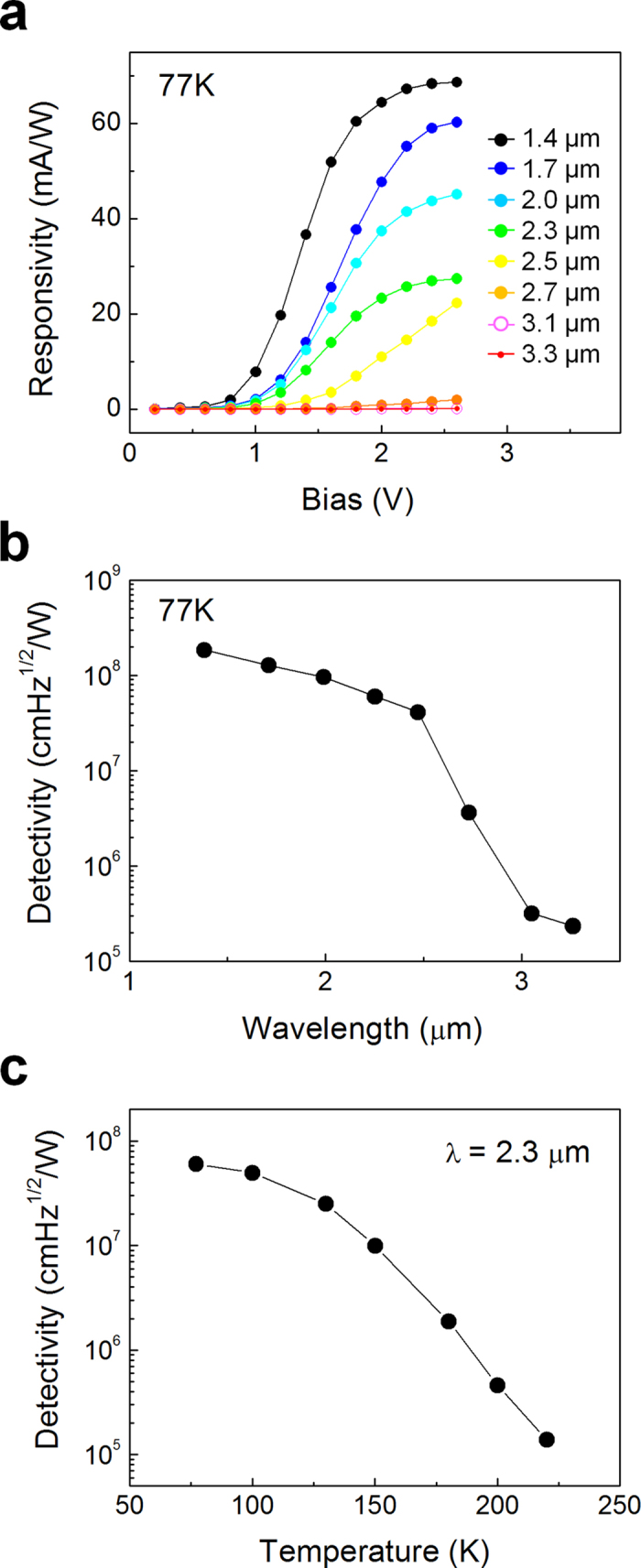
InAs NW array-based photodetector characteristics. (**a**) Responsivity as a function of the measured bias at 77 K. (**b**) Detectivity as a function of the measured wavelength at 77 K. (**c**) Detectivity as a function of the measured temperature at λ = 2.3 μm.

The optoelectronic properties of a photodetector using the strain-induced InAs NWs were examined. Figure 4a shows the photodetector responsivity measured at 77 K for SWIR wavelengths that were selected using band-pass filters. The responsivity of the photodetector is expressed as follows:


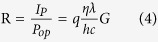


where *P*_*op*_ is the optical incident power, η is the quantum efficiency, λ is the incident wavelength, *h* is Planck’s constant and *c* is the speed of light. Figure 4a shows that the responsivity increased slowly in the low-bias region (V < ~0.8 V). In this region, the photogenerated carriers generally recombine before reaching the electrodes because the drift velocity of the photogenerated carrier is small. In the higher bias range, the responsivity increases rapidly with the bias due to the enhanced drift velocity. Electrons travel much faster in InAs compared to holes. This trend indicates that when the electron transit time is shorter than the minority carrier lifetime, a number of the photogenerated electrons collect at the electrode before they recombine with the holes. This delay results in the electrons re-injecting into the opposite side’s electrode to preserve the charge neutrality of the semiconductor. Therefore, the photoconductive gain of the photodetector increases rapidly with the bias. The gain, however, saturates in the large bias region because the photogenerated holes also reach the electrodes before recombining. The photoconductive gain (*G)* of the photodetector is estimated to be 9.2 × 10^−2^ based on equation (4) with R = 60.45 mA/W, λ = 1.4 μm and η = 59%. The quantum efficiency (η) is calculated from η = (1 − r)(1 − *e*^*−αt*^), where r = 0.01 is the top surface reflection, α = 1.8 × 10^4^ cm^−1^ is the absorption coefficient of InAs at λ = 1.4 μm, and t = 0.5 μm is the device thickness. The device thickness (t) was estimated based on the fact that the total volume of the NW array corresponds to the 0.5-μm-thick planar device in a unit-area. Therefore, a minority carrier lifetime (*τ*) of 0.58 ns is expected from equations (2) and (3) with *l* = 50 μm, *E* = 360 Vcm^−1^ and μ_e_ = 2200 cm^2^V^−1^s^−1^, which is the electron mobility for the strain-induced InAs NWs measured at 100 K^24^. The carrier lifetime of the strain-induced InAs NWs is comparable to that of the Au-catalyzed InAs NWs^25^. Nevertheless, the measured carrier lifetime for the InAs NWs was much shorter than that for high-quality bulk InAs^26^, which is probably due to poor crystallographic quality such as that due to stacking faults^27^. The photodetector detectivity at 77 K is plotted in Fig. 4b as a function of the wavelength. The detectivity, D^*^, which is the figure of merit for photodetectors, is given as follows^28^:


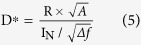


where R is the responsivity, *I*_*N*_ is the measured noise current, Δ*f* = 1 Hz is the measurement system band width, and *A* is the active area. The photodetector detectivity in the SWIR band peaked with 1.9 × 10^8^  cm∙Hz^1/2^/W at λ = 1.4 μm. The NW-based photodetector detectivity decreased with increasing SWIR wavelength, whereas typical InAs photoconductors have peak detectivities at approximately 2.7 μm at 77 K^29^. This difference results from a decrease in light absorption upon increasing the wavelength, as shown in Fig. 2d. Svensson *et al.* reported that the absorption in the SWIR band is strongly related to the NW diameter^30^. Properly selecting the NW diameter can further enhance the NW-based SWIR photodetector detectivity. In Fig. 4c, the photodetector detectivity at λ = 2.3 μm was measured as a function of the temperature. The detectivity decreased from 6.0 × 10^7^ to 1.4 × 10^5^ cm∙Hz^1/2^/W with increasing temperature from 77 to 220 K, respectively. No photocurrent was measured at temperatures above 220 K. The detectivity decreases with increasing temperature because the noise current increases^28^.

In summary, we demonstrated a SWIR photodetector using InAs NWs. Wafer-scale InAs NW arrays were grown on a 2-inch Si substrate via a strain-induced growth method. This densely packed InAs NW array provides excellent AR and light trapping properties over the entire SWIR band. The figure of merit of NW array-based photodetectors that detect the entire SWIR band were measured at temperatures as high as 220 K. We expect various growth techniques, such as inserting a barrier structure^31^ and surface passivation^32^, to significantly enhance the performance of the device. Because Si is extremely cost-effective relative to other semiconductors, InAs NW SWIR photodetectors on Si can be used as future low-cost optical sensors and optical integrated circuits.

## Methods

A metal-organic chemical vapor deposition (MOCVD) system (AIXTRON A200) was used to grow the NWs. A 2-inch Si (111) wafer (ρ = 0.002 – 0.005 Ω·cm) was immersed in a buffered oxide etch for 1 min, and briefly rinsed in deionized (DI) water. Then, the wafer was dipped in poly-L-lysine (Sigma-Aldrich Inc.) for 3 min, cleaned in DI water for 10 s and immediately loaded into the MOCVD chamber. The reactor pressure was pumped down to 50 mbar with a 15 L/min hydrogen flow and heated to a temperature of 570 °C. After a short temperature stabilization time, trimethylindium (TMIn) and arsine gas (AsH_3_) were simultaneously flowed into the reactor. The molar flows for TMIn and AsH_3_ were 2 × 10^−5^ and 2 × 10^−4^ mol/min, respectively. The InAs NWs reached an average height of 50 μm with a growth time of 180 min. Upon completion, the reactor was cooled to a temperature below 300 °C with an AsH_3_ flow.

The total volume (*V*_*tot*_) of the InAs NWs per unit area (*A*) was calculated using the average NW radius (*r*), height (*h*) and number density in unit area (*d*):


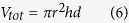


A spectrophotometer system (Cary500Scan, Varian Inc.) was used to measure the reflectance and transmittance spectra of the InAs NW array on Si. A low-doped (ρ = 1 – 10 Ω·cm) and double-side polished Si wafer was used for the optical characterization to minimize free career absorption and light scattering by the Si surface, respectively. For the reflectance, a normal incident light was shone through the InAs NW array on the Si surface, and the reflected light was collected by a detector. For the transmittance, a normal incident light illuminated the planar surface of the Si wafer, and the transmitted light was collected by a detector. An integrated sphere was used to measure the reflectance and transmittance spectra. The wavelength range above 2.5 μm was not measured because of the monochromator’s limit.

To fabricate the photodetector, 50-μm-high InAs NWs grown on Si were spin-coated with cyclotene 3022-57 (BCB, DOW Inc.) at 1500 rpm for 3 min and annealed for 2 hr at 200 °C. The NW tips were exposed slightly via reactive ion etching using CF_4_/O_2_ gas. A 700 nm-thick ZnO film was deposited as a TCO on the exposed NW tips, and then metal electrodes (Ti: 15 nm, Au: 300 nm) were deposited on top of the ZnO, except for an active 2 mm × 2 mm area. The Ti/Au (15/300 nm) were deposited on the back Si surface as back contacts. The fabricated photodetector is shown in Fig. 2a. The fabrication procedure is shown in Figure S2 of the Supplementary Information.

The photocurrent and noise current density of the photodetector were directly measured using a blackbody calibration source (M360, Mikron Inc.), band-pass filters (1.4, 1.7, 2.0, 2.3, 2.5, 2.7, 3.1, 3.3 and 3.6 μm, Spectrogon Inc.) and a lock-in amplifier. The optical incident power (*P*_*op*_) was calculated from the blackbody source using the following relation^28^:


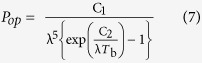


where C_1_ is the first radiation constant (3.7418 × 10^4^ W·μm^4^/cm^2^), C_2_ is the second radiation constant (1.4388 × 10^4^ μm K), λ is the wavelength (μm), and T_b_ is the blackbody source temperature (K). An external power supply (PS2520G, Tekronix Inc.) and low-noise current preamplifier (SR570, Stanford Research Systems Inc.) were used to apply a bias voltage to the photodetector. A lock-in amplifier (7265, Perkinelmer Inc.) was used to measure the photocurrent and noise current density of the photodetector.

## Additional Information

**How to cite this article**: Wook Shin, H. *et al.* Short-wavelength infrared photodetector on Si employing strain-induced growth of very tall InAs nanowire arrays. *Sci. Rep.* **5**, 10764; doi: 10.1038/srep10764 (2015).

## Supplementary Material

Supplementary Information
