# Supplementary material for: Short-wavelength infrared photodetector on Si employing strain-induced growth of very tall InAs nanowire arrays
Source: Sci Rep. 2015 Jun 2;5:10764. doi: 10.1038/srep10764 (PMC4451803; doi:10.1038/srep10764)
Supplement: Supplementary Information [file srep10764-s1.pdf]

Supplementary Information

Short-wavelength infrared photodetector on Si employing strain-induced growth of very tall InAs nanowire arrays

Hyun Wook Shin[1,2,†], Sang Jun Lee[3,†], Doo Gun Kim[1], Myung-Ho Bae[3], Jaeyeong Heo[4], Kyoung Jin Choi[5], Won Jun Choi[6], Jeong-woo Choe[2], and Jae Cheol Shin[7,*]

[1]Korea Photonics Technology and Institute, Gwangju 550-779, Republic of Korea

[2]Department of Applied Physics, Kyung Hee University, Yongin 446-701, Republic of Korea

[3]Korea Research Institute of Standard and Science, Daejeon, 305-340, Republic of Korea

[4]Department of Materials Science and Engineering, Chonnam National University, Gwangju 500-757, Republic of Korea

[5]School of Materials Science and Engineering, Ulsan National Institute of Science & Technology, Ulsan 689-805, Republic of Korea

[6]Korea Institute of Science and Technology, Seoul 136-791, Republic of Korea

[7]Department of Physics, Yeungnam University, Gyeongsan, Gyeongbuk 712-749, Republic of Korea

[†] These authors equally contributed to this work.

[*] Correspondence to: jcshin@yu.ac.kr

KEYWORDS: MOCVD, InAs, Nanowires, Photodetector, SWIR, Silicon

Figure S1 shows the InAs nanowire (NW) array grown on Si (111) substrate under same growth condition but different growth time. More than 99 % of NWs are vertically grown on Si (111) substrate when average NW height is below 10 μm (Figure S1a). However, some portion of the NWs (approximately 10%) is slanted as the NW height reaches 25 μm (Figure S1b). Figure 1c shows that most of NWs (> 90 %) are slightly slanted if their height exceeds 50 μm.

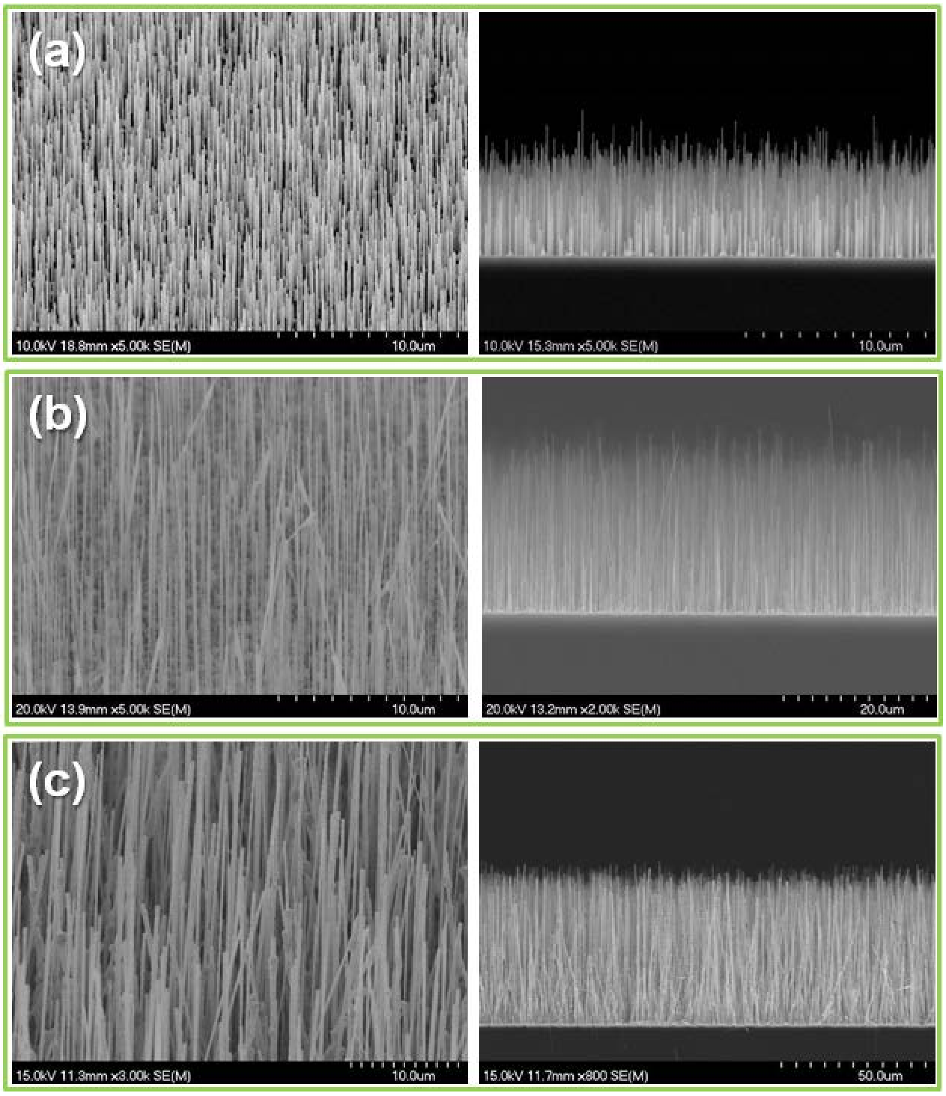

Figure S1. SEM images (left: tilted-view, right: side-view) of InAs NW array grown on Si (111) with different growth time. The average height of the InAs NW array is (a) 6 μm, (b) 25 μm, and (c) 53 μm.

The fabrication procedure of the InAs NW-based SWIR photodetector is detailed in Figure S2.

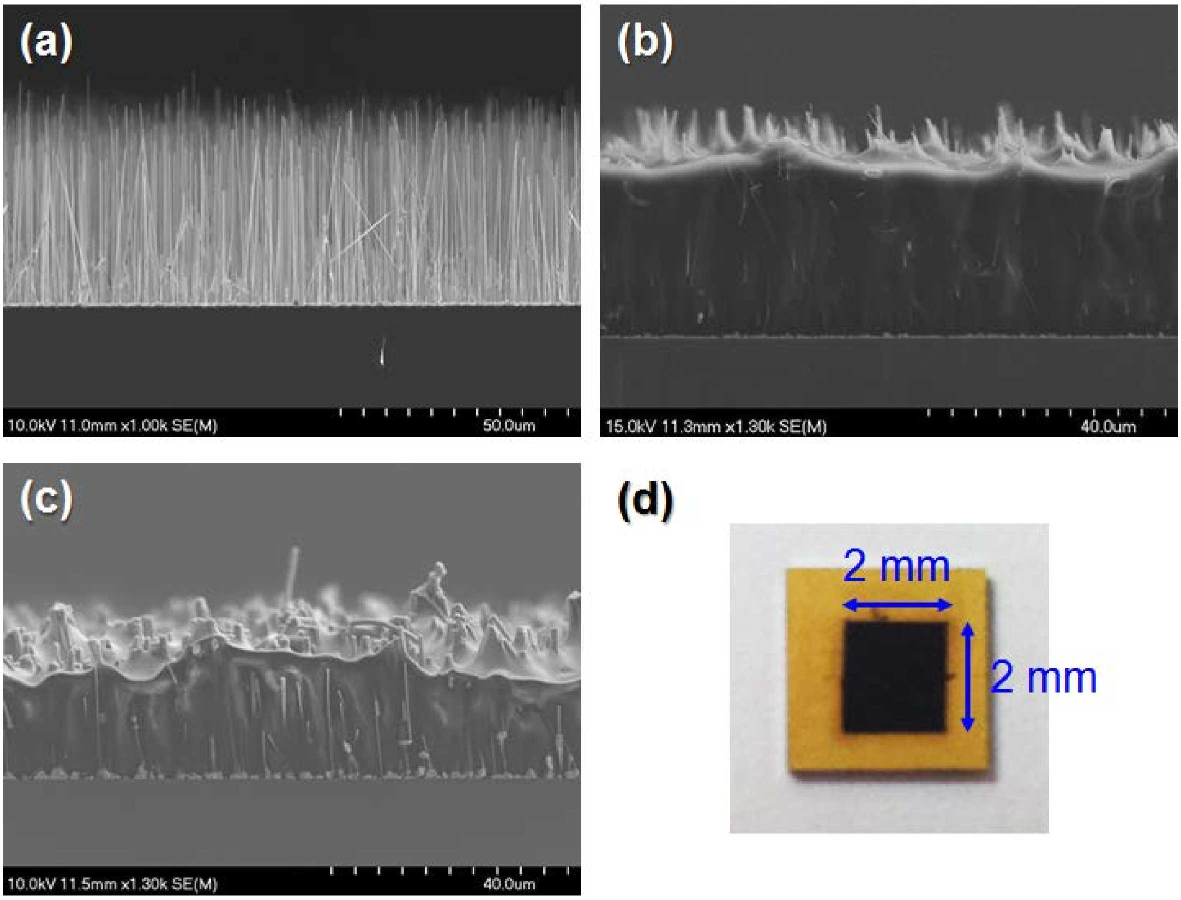

Figure S2. SEM and optical images for the InAs NW-based SWIR photodetector. (a) InAs NW array is grown on heavily n-doped Si (111) substrate. (b) BCB is filled inbetween the NWs. (c) The tips of NWs are exposed by RIE and TCO is deposited on top of the NW array. (d) Top-view optical image of the fabricated photodetector. The active area (2 x 2 mm^2) is defined by open TCO region. Ti/Au is deposited on the rest of area for the top electrode contact. Ti/Au bilayer metals are also deposited on the backside of heavily n-doped Si substrate for the bottom electrode contact.
